# Accuracy, Usability, and Adherence of Smartwatches for Atrial Fibrillation Detection in Older Adults After Stroke: Randomized Controlled Trial

**DOI:** 10.2196/45137

**Published:** 2023-11-28

**Authors:** Eric Y Ding, Khanh-Van Tran, Darleen Lessard, Ziyue Wang, Dong Han, Fahimeh Mohagheghian, Edith Mensah Otabil, Kamran Noorishirazi, Jordy Mehawej, Andreas Filippaios, Syed Naeem, Matthew F Gottbrecht, Timothy P Fitzgibbons, Jane S Saczynski, Bruce Barton, Ki Chon, David D McManus

**Affiliations:** 1 Department of Medicine University of Massachusetts Chan Medical School Worcester, MA United States; 2 Department of Population and Quantitative Health Sciences University of Massachusetts Chan Medical School Worcester, MA United States; 3 Department of Bioengineering University of Connecticut Storrs, CT United States; 4 Department of Pharmacy and Health Systems Sciences Northeastern University Boston, MA United States

**Keywords:** accuracy, atrial fibrillation, cardiac arrhythmia, design, detection, diagnosis, electrocardiography, monitoring, older adults, photoplethysmography, prevention, remote monitoring, smartwatch, stroke, usability

## Abstract

**Background:**

Atrial fibrillation (AF) is a common cause of stroke, and timely diagnosis is critical for secondary prevention. Little is known about smartwatches for AF detection among stroke survivors. We aimed to examine accuracy, usability, and adherence to a smartwatch-based AF monitoring system designed by older stroke survivors and their caregivers.

**Objective:**

This study aims to examine the feasibility of smartwatches for AF detection in older stroke survivors.

**Methods:**

Pulsewatch is a randomized controlled trial (RCT) in which stroke survivors received either a smartwatch-smartphone dyad for AF detection (Pulsewatch system) plus an electrocardiogram patch or the patch alone for 14 days to assess the accuracy and usability of the system (phase 1). Participants were subsequently rerandomized to potentially 30 additional days of system use to examine adherence to watch wear (phase 2). Participants were aged 50 years or older, had survived an ischemic stroke, and had no major contraindications to oral anticoagulants. The accuracy for AF detection was determined by comparing it to cardiologist-overread electrocardiogram patch, and the usability was assessed with the System Usability Scale (SUS). Adherence was operationalized as daily watch wear time over the 30-day monitoring period.

**Results:**

A total of 120 participants were enrolled (mean age 65 years; 50/120, 41% female; 106/120, 88% White). The Pulsewatch system demonstrated 92.9% (95% CI 85.3%-97.4%) accuracy for AF detection. Mean usability score was 65 out of 100, and on average, participants wore the watch for 21.2 (SD 8.3) of the 30 days.

**Conclusions:**

Our findings demonstrate that a smartwatch system designed by and for stroke survivors is a viable option for long-term arrhythmia detection among older adults at risk for AF, though it may benefit from strategies to enhance adherence to watch wear.

**Trial Registration:**

ClinicalTrials.gov NCT03761394; https://clinicaltrials.gov/study/NCT03761394

**International Registered Report Identifier (IRRID):**

RR2-10.1016/j.cvdhj.2021.07.002

## Introduction

Atrial fibrillation (AF) is a common heart rhythm disorder that affects over 6 million Americans and more than 30 million individuals worldwide [1,2]. Individuals diagnosed with AF are at increased risk for myriad adverse health outcomes, including dementia, heart failure, and myocardial infarction [3]. AF is associated with a 5-fold increase in ischemic stroke risk, and AF-related strokes are clinically more severe than those not associated with AF [4]. Oral anticoagulation is highly effective for stroke prevention in AF patients [5]. Unfortunately, nearly one-fifth of the strokes attributable to AF are diagnosed at the time of stroke presentation, highlighting a critical need for improvement in AF detection [6].

Over the last decade, consumer wearable technologies capable of detecting AF have transformed how health care providers and their patients diagnose and manage heart rhythm disorders [7,8]. Several large-scale observational studies, including the Apple Heart Study, Huawei Heart Study, Fitbit Heart Study, and Health eHeart Study, have shown that, in large groups of smartwatch owners, a wearable can offer moderate-to-vigorous accuracy for detecting AF [9-12].

Despite the large number of participants in these studies, relatively few participants were at high risk for AF based on age or comorbidities. Furthermore, all participants were existing smartwatch owners, limiting the generalizability to most older populations in which digital technologies are less commonly used [13,14]. Stroke survivors have a significantly increased risk of having undiagnosed AF than the general population, yet no previous study has examined the use of smartwatches in older stroke survivors [15]. Additionally, stroke survivors often have physical and cognitive impairments, like loss of vision, that may limit smartwatch adoption or impede successful use.

We present the primary findings of the Pulsewatch study (NCT03761394), a randomized trial conducted to evaluate the accuracy and usability of a novel smartwatch-smartphone dyad designed for AF detection among stroke survivors. The Pulsewatch smartphone and smartwatch app were designed on the Android operating system by researchers with significant patient and provider input. The primary aims of this study were to (1) examine the accuracy and usability of a smartwatch for the detection of AF over 14 days compared with a comparator standard electrocardiogram (ECG) patch monitor and (2) describe the extent of adherence to wearing a smartwatch throughout the monitoring period.

## Methods

### Study Population and Setting

All participants were enrolled from ambulatory neurology and cardiovascular clinics affiliated with the University of Massachusetts Memorial Health Care (UMMHC), an academic tertiary care center in central Massachusetts, from September 2019 to August 2021. Ambulatory patients were eligible for participation if they were aged 50 years or older, had a history of ischemic stroke or transient ischemic attack (TIA) within the past decade, were willing to use the Pulsewatch system for at least 44 days, and were proficient in written and spoken English. Exclusion criteria included an absolute contraindication to the receipt of anticoagulation therapy (ie, major intracranial hemorrhage), the inability to provide informed consent, a known allergy or hypersensitivity to medical-grade hydrocolloid adhesives or hydrogel, the presence of a life-threatening arrhythmia that required in-patient monitoring for immediate analysis, and having an implantable pacemaker.

### Study Design

The Pulsewatch study is a clinical trial with 2 phases, both involving randomized assignment of participants to intervention versus control. The first phase is designed to evaluate Pulsewatch system accuracy versus a comparator standard (a 14-day ECG patch) and usability, followed by a second 30-day phase intended to evaluate adherence to wearing the smartwatch daily as the primary study outcome. The Pulsewatch study protocol, including more detailed calculations with regard to sample size and randomization, has been described previously [16].

### Study Procedures

Trained research staff screened electronic medical records for eligible patients with upcoming neurology and cardiology clinic appointments from September 2019 to May 2021. An invitation letter providing a description of the Pulsewatch study as well as contact information was mailed to all participants in case they wished to ask questions or opt out of the study. Patients were then approached in person by research coordinators at the time of their ambulatory clinic visit to gauge interest in the study and obtain informed consent, if appropriate. Upon enrollment, participants were asked to complete a baseline questionnaire and were then randomized to either the control or intervention groups in a 1:3 ratio. The random allocation sequence was generated a priori by statisticians, to which all other study staff were blinded. Both groups received a comparator standard US Food and Drug Administration (FDA)–cleared ECG patch (Cardea Solo, Cardiac Insight) and were asked to wear the patch daily over 14 days, whereas participants in the intervention group received the same ECG patch and instructions for use plus a Pulsewatch system (Samsung Gear S3 or Galaxy Watch 3 with accompanying Samsung smartphone). Participants were asked to wear the smartwatch daily and to keep the smartwatch and smartphone charged regularly. Research staff provided training to all intervention group participants, as well as to any caregiver or family member who accompanied the participant to the study visit, and all participants were provided a training packet with detailed instructions for the successful use of the patch and the Pulsewatch system (Multimedia Appendix 1).

During the 14-day follow-up period for phase 1 of the study, research staff contacted participants on days 3 and 7 to encourage the use of the Pulsewatch system, address any questions or concerns expressed by participants, and troubleshoot any technical challenges the participant may be experiencing. This training was designed to improve wear time and ensure adequate sampling to evaluate the accuracy of the Pulsewatch system.

**Figure 1:**
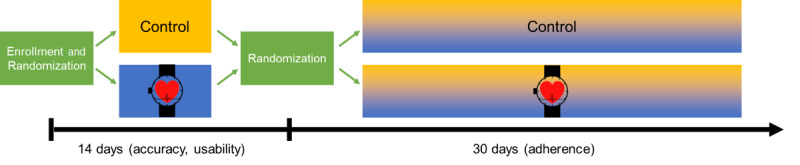
Pulsewatch study design and randomization process.

Upon completion of the 14-day follow-up period, participants returned for a study visit, at which time they completed a follow-up questionnaire to assess key domains, including device use experience and a multitude of psychosocial factors, and those in the intervention arm were asked about their experience with the smartphone, app, and smartwatch. At this time, all participants, irrespective of their initial assignment to intervention or control, were randomized in a 1:1 fashion to be provided the Pulsewatch system for an additional 30 days. This 30-day follow-up period was designed to evaluate longer-term adherence to smartwatch use. During this phase of the study, the research staff did not initiate any calls with participants. The staff did, however, provide advice and instructions to participants who called the study hotline during the follow-up period. An overview of the study design is presented in Figure 1.

### Pulsewatch System

The Pulsewatch system consists of 2 apps developed for the Android operating system for use on the Samsung smartphone and smartwatch. The Pulsewatch user interface was designed through an iterative process involving participants who consented to participate in focus groups and a Hack-a-thon. Participants included stroke survivors, their caregivers, and health care providers. Development and programming took place over the course of a year, and 4 focus groups were held with patients and their caregivers. During these sessions, iterations of the app interface were presented to the participants to elicit their feedback and refine the app for better usability.

Upon completion of the focus groups, additional patients and their caregivers, together with local cardiologists, cardiac electrophysiologists, stroke neurologists, and the primary engineering and app development teams, were invited to a day-long programming event where participants reviewed the prototype app, offered their perspectives, and made suggestions for improvement.

The final version of the Pulsewatch smartwatch app shows the user their heart rate, the time, and their rhythm status based on the recordings of the participants’ pulse (5-minute recordings are used to assess rhythm). If at least 90 consecutive seconds of potential AF are detected during a 5-minute detection window, the watch app displays an alert asking the user to “please stay still” along with an accompanying audio or vibration notification. After this alert, if another 60 consecutive seconds of AF are detected, the user is presented with an alert on the watch that reads “abnormality detected.” This approach was designed to reduce the likelihood of motion noise artifact simulating AF and the number of false positives among participants. “Abnormality detected” was our primary outcome, indicating Pulsewatch’s detection of AF.

**Figure 2:**
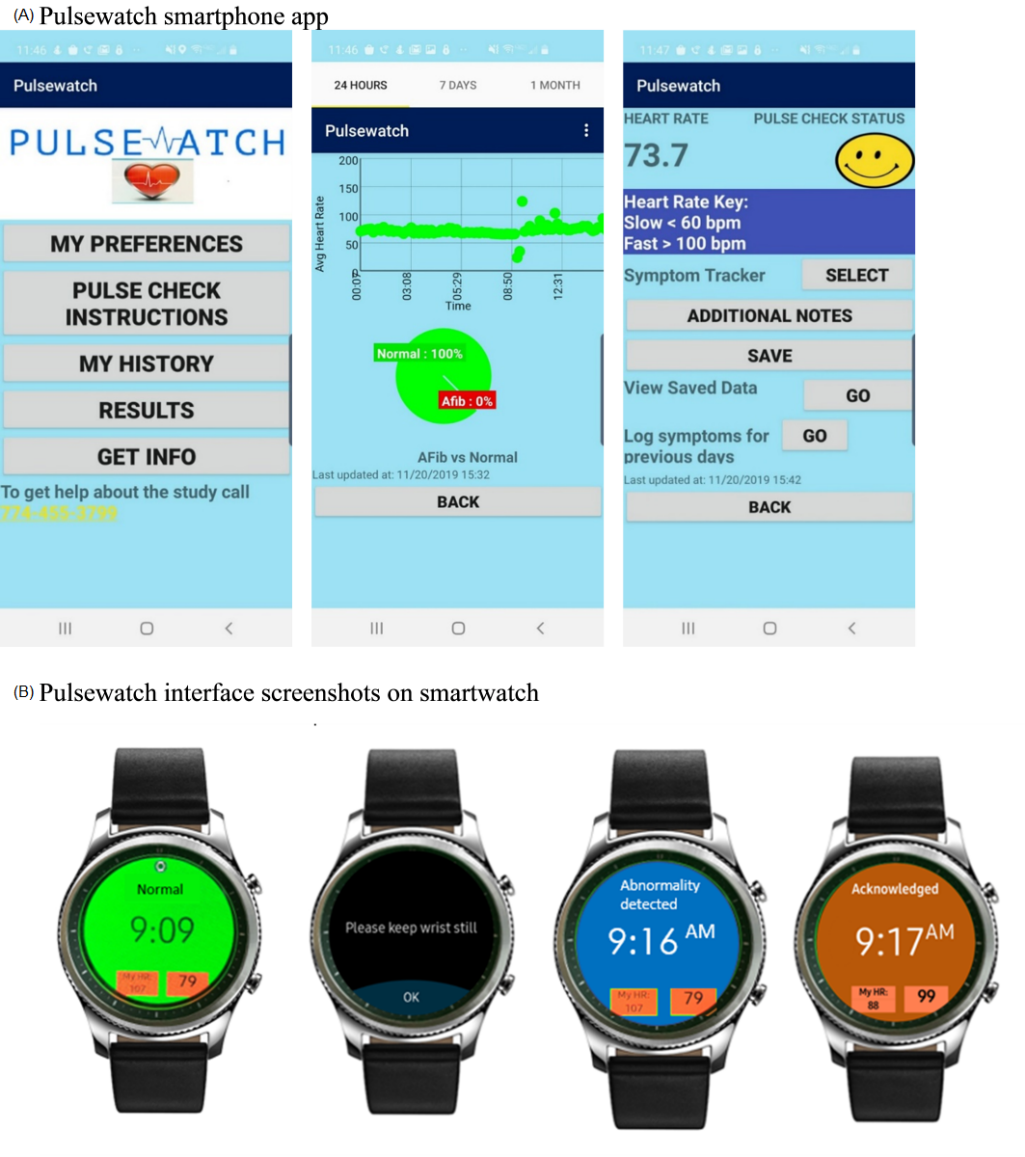
Representative screenshots of Pulsewatch apps.

The Pulsewatch smartphone app pulls pulse data from the smartwatch, allows users to label arrhythmia episodes with any symptoms they may be experiencing, provides informational links regarding AF, and allows participants to review their heart rate ranges. Screenshots from the final version of the smartphone and smartwatch apps are presented in Figure 2.

### Study Measures and Primary Outcomes

Participant demographic characteristics and medical history information were abstracted from the electronic medical record by trained research staff. Accuracy of the smartwatch’s “abnormality detected” (or AF detection) was determined by comparing whether a participant received any AF alerts over the 2-week monitoring period compared to whether AF was adjudicated as present based on a cardiologist’s over-read of all possible AF episodes detected on the FDA-cleared ECG patch monitor. Raw ECG data from these patches were extracted, and each episode categorized as AF by the patch monitor’s built-in FDA-approved AF detection algorithm was divided into 30-second segments. Each 30-second segment was reviewed by a noncardiologist physician (SN), and those determined to be potentially AF were reviewed by a board-certified cardiologist (MFG) for confirmation. Any segments on which the reviewers disagreed were reviewed by another board-certified cardiologist (KVT) as a tiebreaker. If any participant failed to wear the Pulsewatch smartwatch during the 14-day study period, they were excluded from the device accuracy analysis.

Usability was measured at the 14-day study examination by the System Usability Scale (SUS), a validated scale ranging from 0 to 100, with scores of 68 or greater indicating high usability, and investigator-generated Likert scale usability questions specific to smartwatch use [17]. Pulsewatch system adherence was defined by the number of hours of daily smartwatch wear time over the 30-day monitoring period. Participants were considered to have worn the watch during a given hour if they had either more than 50 recorded steps or any heart rate data recorded during that hour. Mean wear time was calculated for each day of the study, and daily adherence was operationalized in 2 ways: wearing the watch for at least 1 hour and wearing the watch for at least 5 hours.

### COVID-19 Protocol Adaptations

Due to the unprecedented challenges presented by the COVID-19 pandemic, this study protocol was adapted in June 2020 and approved by the Institutional Review Board to be performed entirely through remote means to ensure the safety of study participants, research staff, and clinic staff. All in-person study visits were replaced by phone encounters, including informed consent, enrollment, baseline visit, and follow-up visits for both phases of the study. All participants approached after June 2020 were given the option of interfacing with study staff in person per the original study protocol or entirely remotely as described above. The only questionnaire that could not be administered over the phone as it was in person was the Montreal Cognitive Assessment (MoCA) [18]. A validated version of the MoCA designed for administration through telephone was administered to assess patients’ cognitive status as part of our COVID-19 protocol [19].

### Data Management

Deidentified pulse data collected from the smartwatches were transferred to the paired smartphone through Bluetooth in real time, and these data were transmitted to secure study servers for storage. All patient identifiers, questionnaire data, and information extracted from medical records were stored on Health Insurance Portability and Accountability Act (HIPAA)–compliant secure servers at UMMHC.

### Data Analysis

Descriptive statistics were calculated for all participant demographic, medical history, and psychosocial characteristics. The performance of the Pulsewatch system for AF detection was determined at the individual participant level and presented as sensitivity, specificity, and accuracy compared to the comparator standard. For usability questions, Likert scale responses of “strongly agree” and “agree” were collapsed, as were responses of “strongly disagree” and “disagree.” Descriptive statistics of usability questions were calculated. Overall device wear time in hours was quantified for each participant and descriptive statistics were calculated. A Cuzick rank sum test for the trend of ordered groups was used to assess the linear trend in daily adherence (binary outcome). All analyses were completed using Stata 14.0 and SAS 9.3.

### Ethics Approval

Pulsewatch has been approved by the UMass Chan IRB (Approval Number H00016067). Written informed consent was collected from all participants, and all study data has been deidentified. Study participants were compensated US $100 for their participation in phase 1, then another US $100 for phase 2. A small convenience sample of participants was selected for qualitative interviews about their experience in the study and compensated US $60 for their time.

## Results

### Overview

A total of 90 participants were randomized into the intervention arm of phase 1 of the trial and 57 into the phase 2 intervention group. The average age of study participants in the first phase was 65.1 (SD 9.3) years, 41% (37/90) were female, most were non-Hispanic White (78/90, 87%), 56% (46/82) were college graduates, and 35% (27/77) had an income higher than US $100,000. More than 3 out of 4 participants had been previously diagnosed with hypertension and hyperlipidemia, and 2 out of every 5 were cognitively impaired. Most owned smartphones (74/89, 83%) and engaged with apps on their devices daily (54/80, 68%), though a much smaller proportion of participants owned smartwatches (22/89, 25%). The characteristics of participants randomized into the phase 2 intervention group were similar. Participant characteristics are further detailed in Tables 1 and 2.

**Table 1:** Baseline medical characteristics of study participants randomized to use the Pulsewatch smartphone app and smartwatch.

Demographics		Phase 1 (n=90)	Phase 2 (n=57)
Age (years), mean (SD)		65.1 (9.3)	64.1 (8.8)
Female, n (%)		37 (41)	22 (39)
**Race, n (%)**			
	White	78 (87)	52 (91)
	More than one race	6 (7)	2 (4)
	Black	1 (1)	1 (2)
	Asian or Pacific Islander	1 (1)	1 (2)
	Other	4 (4)	1 (2)
Non-Hispanic ethnicity, n (%)		87 (97)	57 (100)
Married or living as married, n (%)		61 (69)	37 (66)
**Education, n (%)**			
	Less than high school	4 (5)	3 (5)
	High school degree or equivalent	38 (43)	24 (44)
	College degree	28 (32)	15 (27)
	Postgraduate degree	18 (20)	14 (25)
**Income (US $)**			
	Less than 50,000	29 (35)	15 (29)
	Between 50,000 and 99,999	27 (33)	18 (34)
	More than 100,000	27 (33)	19 (37)
**Medical history, n (%)**			
	History of ischemic stroke	71 (79)	46 (81)
	History of TIA^a^	26 (29)	18 (32)
	Congestive heart failure	6 (7)	4 (7)
	Cardiac arrhythmias	12 (13)	8 (14)
	Valvular disease	9 (10)	9 (16)
	Hypertension	70 (78)	41 (72)
	Chronic pulmonary disease	7 (8)	7 (12)
	Diabetes	25 (28)	7 (12)
	Vascular disease	24 (27)	13 (23)
	Renal disease	4 (4)	3 (5)
	Previous major bleed	5 (6)	5 (9)
	Previous MI^b^	16 (18)	10 (18)
	Hyperlipidemia	77 (86)	48 (84)
	Sleep apnea	25 (28)	14 (25)
	Percutaneous coronary intervention	11 (12)	6 (11)
**Medication use, n (%)**			
	Antiarrhythmic	2 (2)	1 (2)
	Beta blocker	40 (44)	19 (33)
	Calcium channel blocker	62 (69)	42 (74)
	Anticoagulant	11 (12)	12 (21)
	Antihypertensive	51 (57)	31 (54)
	Antiplatelet	79 (88)	45 (79)
	Statin	82 (91)	53 (93)
**Vitals, mean (SD)**			
	BMI	32 (21)	30 (10.3)
	Systolic BP^c^	131.4 (16.7)	129.4 (15.2)
	Diastolic BP	76 (8.6)	75.8 (9.4)
	Heart rate	73.1 (14.7)	74.8 (13.9)

^a^TIA: transient ischemic attack.

^b^MI: myocardial infarction.

^c^BP: blood pressure.

**Table 2:** Psychosocial characteristics of study participants randomized to use the Pulsewatch smartphone app and smartwatch.

Characteristics				Phase 1, n (%)		Phase 2, n (%)
Residual neurological deficit				28 (31)		17 (30)
Alcohol use				7 (8)		5 (9)
Cognitive impairment^a^				26 (30)		19 (34)
Vision impairment				48 (54)		23 (40)
Hearing impairment				26 (29)		16 (28)
**Depressive symptoms**						
	None (0-4)		49 (54)		32 (56)	
	Mild (5-9)		27 (30)		19 (33)	
	Moderate (10-14)		8 (9)		3 (5)	
	Moderately severe (15-19)		3 (3)		2 (4)	
	Severe (>20)		3 (3)		1 (2)	
**Anxiety symptoms**						
	None or minimal (0-4)		62 (69)		37 (65)	
	Mild (5-9)		16 (18)		12 (21)	
	Moderate (10-14)		7 (8)		6 (11)	
	Severe (>15)		5 (6)		2 (4)	
**Technology engagement**						
	**Device ownership**					
		Tablet	60 (67)		42 (74)	
		Smartphone	74 (83)		48 (84)	
		Smartwatch	22 (25)		18 (32)	
		Basic cellphone (SMS-enabled)	30 (34)		14 (25)	
	**App use frequency (excluding call or text)**					
		Daily	54 (68)		36 (72)	
		A few days a week	12 (15)		4 (8)	
		At least once a week	5 (6)		4 (8)	
		Less than once a week	2 (3)		1 (2)	
		Once a month	3 (4)		2 (4)	
		Never	4 (5)		3 (6)	

^a^Montreal Cognitive Assessment (MoCA) <23 for in-person, <17 for phone version.

### Atrial Fibrillation Burden

AF was detected in 6 out of the 90 participants (incidence 6.67%). Participants with AF detected varied widely with regard to the number of episodes and the total AF burden (time spent in AF), ranging from a minimum of 2 episodes lasting a total of 16 minutes to 5012 episodes lasting a total of 1712 minutes (Table 3). However, despite this wide variation in total AF, the length of the longest episode was less than 30 minutes in duration (mean 14.6, SD 8). Notably, 1 participant did not wear the watch at all and was thus excluded from subsequent accuracy analysis.

**Table 3:** Burden of atrial fibrillation (AF) detected in participants in Pulsewatch.

Participant ID	AF episodes, n (%)	Total AF burden (minutes)	Longest AF episode (minutes)
005	2 (0)	16	9.6
017	400 (2)	451	20.5
026	1133 (3)	322	5
051	5012 (15)	1712	10.1
075	215 (5)	426	26.8
082	9 (0)	52	15.7

### Accuracy

The Pulsewatch system detected AF correctly in 3 out of the 5 participants who were determined to have AF by cardiologist overread of the ECG patch monitors (60% sensitivity, 95% CI 14.7-94.7) and correctly indicated no AF in 76 of the 80 participants determined to be free from AF based on cardiologist overread of the ECG patch monitors (95% specificity, 95% CI 87.7-98.6). Overall, the smartwatch exhibited an accuracy of 92.9% for AF identification in our 14-day study (Tables 4 and 5).

Although a participant may be wearing the watch, the Pulsewatch app may not have been constantly running in the background, most often due to accidental termination of the app by the participant or activation of power-saving mode to conserve battery. Thus, active rhythm recording did not occur for the entirety of watch wear time for all participants. Accuracy analysis is therefore limited to the duration that the Pulsewatch app was on and recording pulse (Table 3).

**Table 4:** Smartwatch-detected atrial fibrillation (AF) compared with an electrocardiogram (ECG) patch over 2-week monitoring period.

		ECG patch, n			Total
		AF	No AF		
**AF alerts on smartwatch, n**					
	Alerts	3	4	7	
	No alerts	2	76	78	
Total		5	80	85	

**Table 5:** Accuracy of smartwatch-detected atrial fibrillation (AF) compared with an electrocardiogram (ECG) patch.

Statistic	Value (%) (95% CI)
Sensitivity	60 (14.7-94.7)
Specificity	95 (87.7-98.6)
Positive predictive value	42.9 (18.5-71.2)
Negative predictive value	97.4 (92.8-99.1)
Accuracy	92.9 (85.3-97.4)

### Usability

The average Pulsewatch SUS was 62.8 out of a possible 100, and 37.5% (33/88) of participants reported the system as highly usable (SUS ≥68). When asked about the watch and phone apps separately, 63.6% (56/88) of participants agreed or strongly agreed that the watch app was easy to use, while 52.3% (46/88) indicated the same for the phone app. Around 42% (37/88) of participants agreed or strongly agreed that using the Pulsewatch system made them feel more connected to their doctor. Most participants did not experience anxiety or worry because of using the Pulsewatch system (60/88, 68.2% disagreed or strongly disagreed), and 58% (51/88) of participants indicated a willingness to use the system daily for 6 months for heart rhythm monitoring. Overall, more than one-half of participants, 51.1% (45/88), agreed or strongly agreed with the statement that they enjoyed using the system over the course of the study. All 90 participants who used the Pulsewatch system indicated that they would be comfortable allowing their doctors access to health information collected by this device.

### Participant Adherence to Smartphone App and Smartwatch Use Over the 30-Day Phase 2 Period

**Figure 3:**
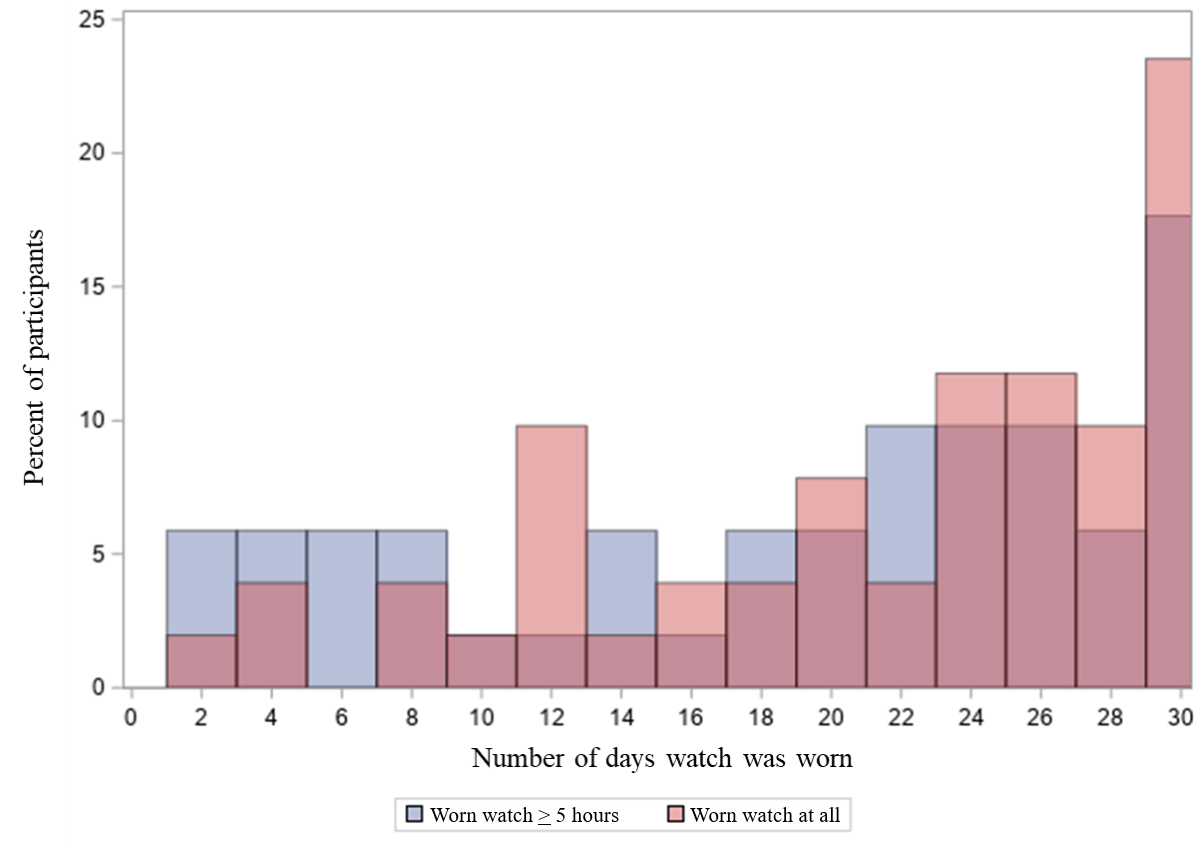
Distribution of days of watch wear over course of study.

**Figure 4:**
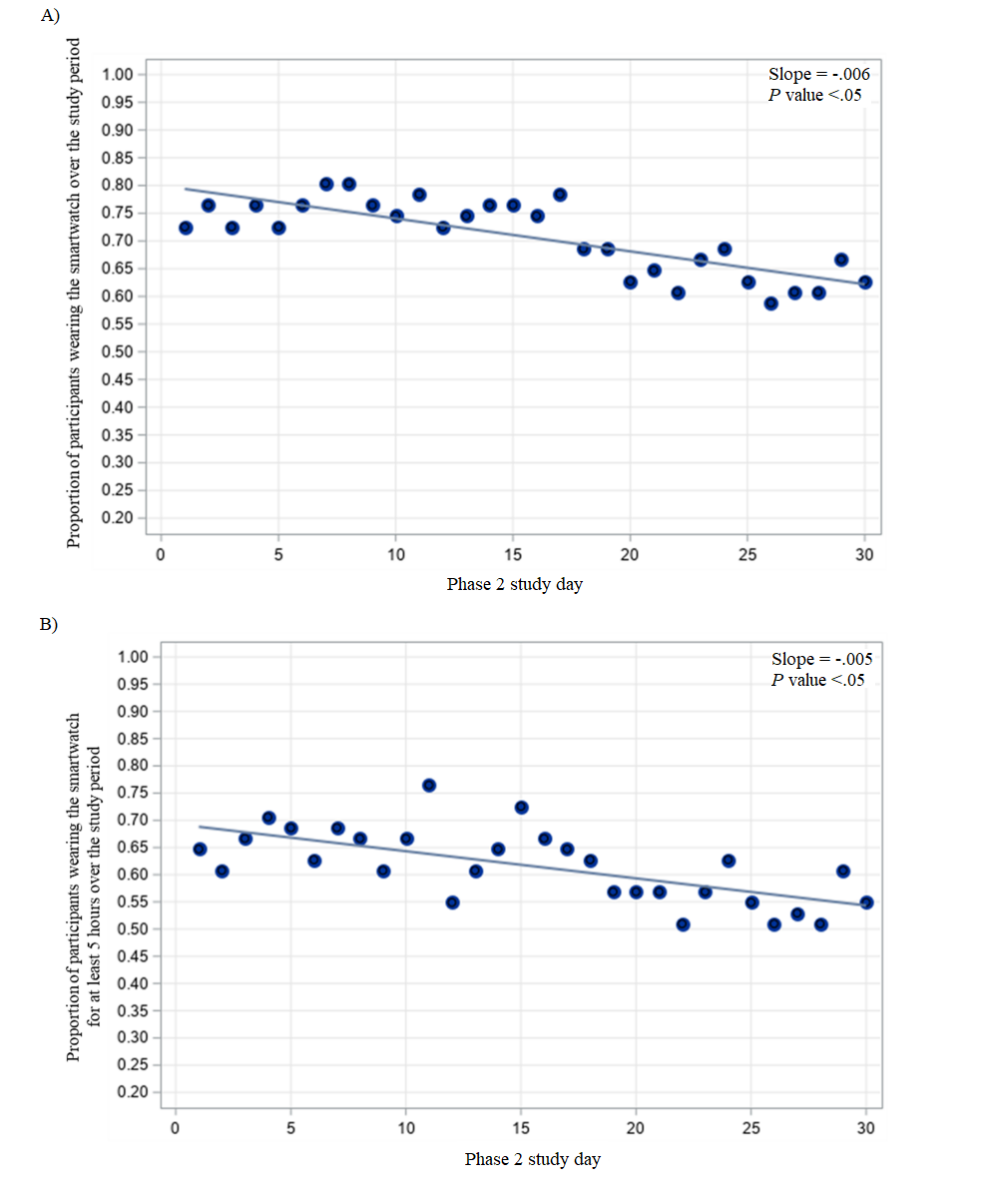
Proportion of participants wearing the Pulsewatch smartwatch (A) at all or (B) over 5 hours on each day of the 30-day phase 2 follow-up period.

During the second phase of the study, participant adherence remained steady overtime (Figure 3). Initially, 73% (37/51) of participants wore the watch, while on day 30, this slightly decreased to 63% (32/51) of participants (Figure 4A) (*P*<.05). Similarly, about 55% (28/51) of participants wore the watch for at least 5 hours on day 30 of the study (Figure 4B). Participants wore the watch for 21.2 (SD 8.3) days (out of the 30 possible days), but they kept the watch on for the majority of waking hours during those days (average 11.5, SD 5.1 hours).

## Discussion

### Overview

In this manuscript, we describe the accuracy and usability of a novel smartphone-app smartwatch system for detecting undiagnosed AF designed with and for stroke survivors. Compared to a gold-standard cardiologist’s over-read of ECG patch recordings, the Pulsewatch app and smartwatch dyad demonstrated 93% accuracy at the participant level over the 2-week monitoring period. Approximately half of the participants found the Pulsewatch system to be highly usable, and over half of the participants reported that they would be interested in using the smartphone-app smartwatch for AF monitoring after study completion. In contrast to previous studies, we observed a slight decline in adherence to watch wear over 30 days, with several participants opting to keep the watch on after the study [20]. Our findings suggest that a smartphone app and smartwatch system for AF detection is accurate and has reasonable participant adherence to watch wear to serve as a clinical method of AF detection. However, the slight decline in adherence observed in this study suggests that clinical deployment of smartwatches for AF detection may benefit from further development of strategies to enhance adherence to watch wear.

### Accuracy of the Smartphone App-Smartwatch Dyad (Pulsewatch System) for Atrial Fibrillation Detection

In this randomized study of older stroke survivors, we observed high patient-level accuracy in detecting undiagnosed AF. The incidence of AF in the Pulsewatch cohort was relatively high during the 14-day follow-up (6.67%) compared to similar cohorts. A meta-analysis of 50 studies examining AF diagnosis poststroke demonstrated a yield of 15.3% with mobile cardiac outpatient telemetry (MCOT) devices [21]. However, AF incidence in this study is still within the confidence interval (5.3%-29.3%). Furthermore, Pulsewatch included participants without diagnosed AF whose stroke occurred up to a decade ago. Thus, most individuals in our cohort have undergone extensive work-up post stroke to identify AF as a potential source of their stroke, thus any arrhythmia we identify is occult AF that has been refractory to detection up to this point, in some cases many years after their stroke. This is further exemplified by the relatively low burden of AF among all participants in whom AF was detected and the longest episode being 27 minutes.

The Pulsewatch algorithm for AF detection was designed to minimize false-positive alerts to avoid introducing patient anxiety. This is particularly important in the context of our recent finding that smartwatch alerts for AF negatively impact patients’ perceptions of physical health [22]. In this study, we developed a patient-centric smartwatch monitoring system that has high negative predictive value and low false positives, demonstrating a viable option for AF detection that does not cause undue stress due to excessive false positive alerts.

Our analysis of concurrent watch and patch wear demonstrated that while a portion of the hours in which AF was detected on the patch were not detected by the smartwatch, it was still able to identify AF in 3 of the 5 participants who had this condition over the 2-week monitoring period. This further highlights the importance of adequate duration of monitoring, which may be significantly higher for a smartwatch than a traditional ECG monitor affixed to a patient’s chest, as a smartwatch may need to analyze more episodes of AF to detect the condition, as observed in this study. We observed that 55% (28/51) of participants in this study wore the watch for greater than 5 hours daily at 30 days, suggesting that in real-world use, smartwatches would be worn for an adequate duration to allow for AF detection, albeit with a slight decline in adherence over time.

Few studies have examined the accuracy of wearable devices among older adults, and fewer still among those naïve to technologies at baseline. The Apple Heart Study recruited individuals who already owned an Apple Watch (average age 41 years, 57% male), asked them to use the device’s built-in pulse analysis feature, and sent ECG patch monitors to all participants who received a potential AF alert on their smartwatch [9]. Among the 450 users who returned their ECG patches, 34% had AF identified on their clinical gold-standard patches, and participants who were of age 65 years or older had the highest rate of AF identified. The Huawei Heart Study used a similar design to that of the Apple Heart Study, and of the 262 participants with “suspected AF” notifications on their smartwatch who had subsequent clinical workups, 87% had confirmed AF [10]. Similarly, the study found that older adults had the highest rate of AF being confirmed as present among those who received alerts on their watches. While neither of these studies specifically addressed smartwatch use among older adults or patients with stroke, they reinforce the high incidence of AF and illustrate the potential for wearables to detect undiagnosed AF that is clinically significant. Fortunately, there are ongoing efforts to focus on this understudied but high-risk population. The Liverpool-Huawei stroke study is an ongoing prospective study that plans to enroll 1000 stroke patients and monitor them for 4 weeks using a Huawei wearable device [23]. Eligibility criteria are quite similar compared to Pulsewatch, and it will be quite interesting to compare the incidence of AF between the studies.

### Perceptions of Smartwatch Usability

Older adults are a particularly understudied population with respect to digital health and telemedicine, and information on the perceptions of smartwatch usability among this population is scant. A systematic review of wearable sensors deployed in older adults found that studies largely focus on the systems and technical performance aspects of technology and that few existing studies address usability and acceptability challenges encountered by older adults [24].

A preliminary feasibility study we conducted in a small cohort of older adults (n=40, mean age 71 years) using a smartwatch for cardiac rhythm monitoring found an average SUS score of 73 [13]. In the general population, SUS scores for various smartwatch models are typically in the range between 60 and 70, depending on the specific model or app scenario [25,26]. Furthermore, a usability study of older adults (aged between 66 and 88 years) using the Samsung Galaxy Gear S3 to administer assessments of pain, mood, and fatigue showed that 73% of participants reported being satisfied with the smartwatch, and another 73% reported that they were likely to use the device daily in the context of a research study for a year [27]. Similarly, a qualitative study of 19 adults over the age of 65 years with osteoarthritis found that 74% of participants indicated a willingness to use a smartwatch for a year for pain symptom tracking [28]. Pulsewatch participants were generally less amenable to long-term device engagement (60% of participants indicated a willingness to use the system daily for 6 months), but this difference may stem from differences in the populations under study and their reasons for smartwatch use. Both previous studies focused on using smartwatches for pain assessment in patients with osteoarthritis, a condition that can drastically diminish quality of life and thus may be perceived by participants as a health priority more so than heart rhythm monitoring [28]. Overall, our data is promising with regard to the long-term use of smartwatches as a viable option for rhythm monitoring with regard to perceived usability.

### Adherence to Smartwatch Use

Initial adherence to the Pulsewatch app and smartwatch was high but declined marginally over the course of the 30-day monitoring period. Our findings are consistent with other studies that prescribe the use of commercial wearables among older adults with risk factors for stroke. For example, in a substudy of the mHealth Screening to Prevent Strokes (mSToPS) trial, which consisted of 230 participants with numerous risk factors for AF (median age 71 years; 24% female), it was observed that 43% of participants who received a smartwatch to monitor their heart rhythm did not record any data on the device despite initial enthusiasm for receiving such a device [29]. The distribution of device usage over a follow-up period of 4 months in mSToPS showed that 43% of participants never transmitted any data. In this study, all participants wore the watch for at least 2 days; more than one-fifth wore the watch at least once a day, and over a tenth wore it for more than 5 hours a day for the entirety of the 30-day study period. This is likely a direct result of the highly specialized and intensive training Pulsewatch participants received at the initial study visit, along with on-call technical support, which was not part of the protocols for previous studies. This further highlights the importance of personnel support when deploying wearable devices in older adults, as though it is extremely resource-intensive, the level of support required to address barriers to device use in this population is high.

Our results illustrate the opportunities for using wearables for AF detection but also highlight gaps that may impede the successful integration of smartwatches for long-term arrhythmia monitoring in at-risk populations that do not regularly use such devices. As discussed in the recent European Heart Rhythm Association (EHRA) position paper on AF screening and detection, photoplethysmograph-based wearables potentially provide a useful means of initial screening but should not guide anticoagulation therapy without further investigation [30]. Since the performance of the system for AF monitoring is predicated on adherence to watch wear, our observations suggest that systems must be refined and supported for them to perform as well as implantable monitors or other devices designed for longer-term arrhythmia monitoring. Further research is needed to identify whether certain baseline characteristics (eg, familiarity with technology, smartwatch ownership, marital status, or social support) may identify populations better able to use prescribed smart devices over longer periods. Furthermore, with the rise in holistic, integrated AF treatment centers that focus on behavior change and healthy lifestyle promotion, research into whether support from clinical teams can reengage and sustain wearable use for AF monitoring is necessary [31]. The “Atrial Fibrillation Better Care” (ABC) pathway introduced by the mAFA-II and Huawei Heart Study investigators is a prominent example of the potential of well-designed and executed models, achieving excellent outcomes in terms of anticoagulation rates for patients diagnosed with AF [32].

### Limitations

Our results should be examined in light of several limitations. First, our small sample size and subsequent low rate of AF result in large confidence intervals for the accuracy of AF detection, and thus the accuracy values should be interpreted with this in mind. Additionally, watch wear time in the study was relatively low and may have resulted in inadequate coverage to capture AF in subjects with arrhythmia, further complicating accuracy calculations. Finally, this study population is rather homogenous, including a high proportion of individuals of high socioeconomic status, which may not be representative of other populations.

### Conclusions

A smartphone-app smartwatch dyad designed with and for patients with a previous stroke demonstrates high accuracy for detecting undiagnosed AF and was found to be highly usable by stroke survivors. Daily adherence to the system declined over a 30-day unsupported monitoring period, suggesting that the use of commercial wearables for AF detection outside of populations who previously owned such devices will require new strategies to improve adherence for effective integration of wearables into clinical settings.

## Appendix

Multimedia Appendix 1 Supplemental materials and information regarding the Pulsewatch RCT.

Multimedia Appendix 2 CONSORT diagram detailing study enrollment and retention.

Multimedia Appendix 3 CONSORT-eHEALTH checklist (V 1.6.1).

## Abbreviations

ABC: Atrial Fibrillation Better Care
AF: atrial fibrillation
ECG: electrocardiogram
EHRA: European Heart Rhythm Association
HIPAA: Health Insurance Portability and Accountability Act
MCOP: mobile cardiac outpatient telemetry
MoCA: Montreal Cognitive Assessment
mSToPS: mHealth Screening to Prevent Strokes
RCT: randomized controlled trial
TIA: transient ischemic attack
UMMHC: University of Massachusetts Memorial Health Care

## Data Availability

The data sets generated during or analyzed during this study are not publicly available. Please contact the corresponding author for inquiries regarding data access.
