# Supplementary figures and images for: Accuracy, Usability, and Adherence of Smartwatches for Atrial Fibrillation Detection in Older Adults After Stroke: Randomized Controlled Trial
Source: JMIR Cardio. 2023 Nov 28;7:e45137. doi: 10.2196/45137 (PMC10716742; doi:10.2196/45137)

**Figure S1.** Atrial fibrillation (AF) detected in Pulsewatch study.**
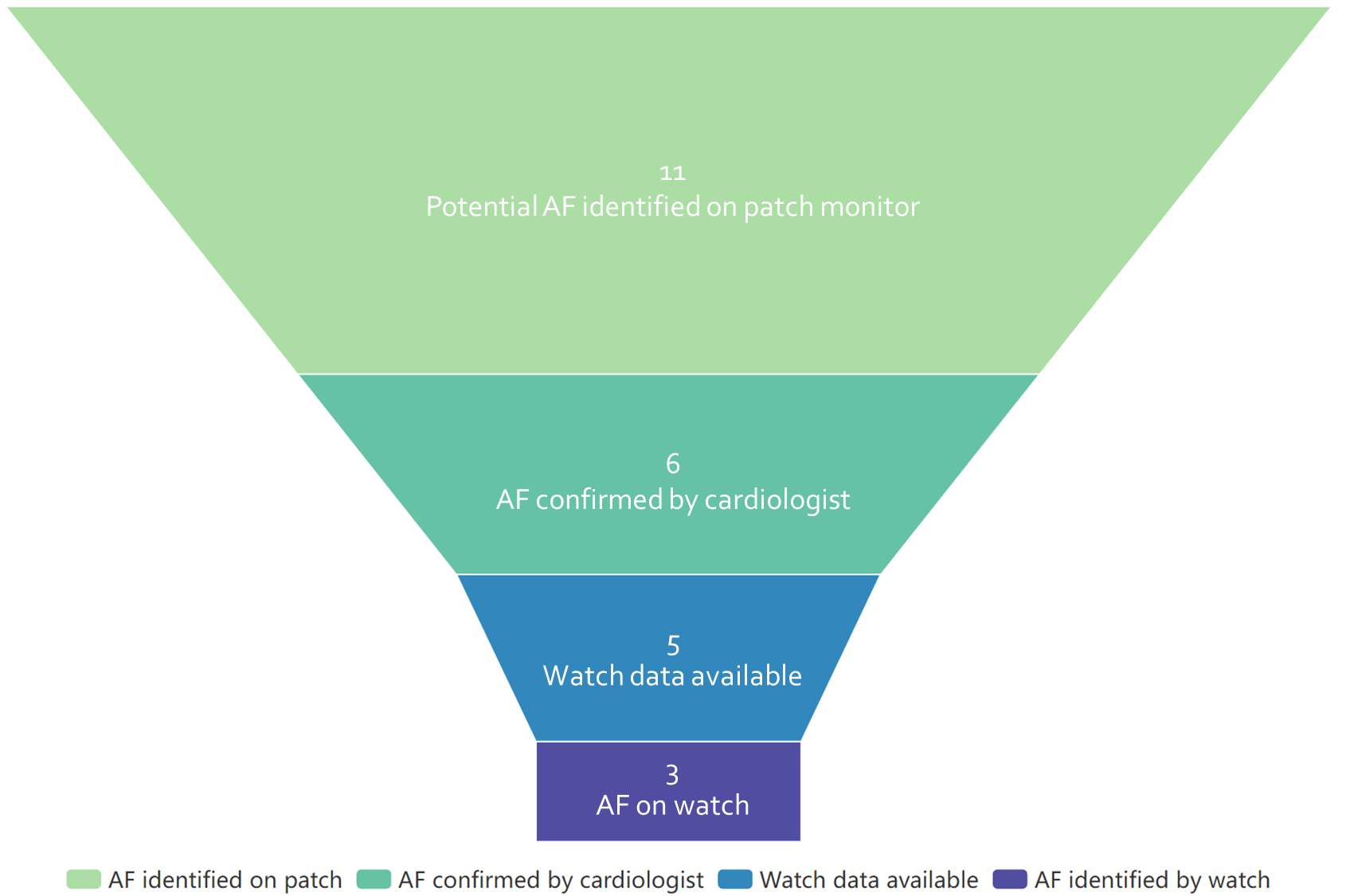
**

**User guide for participants:**

Supplement: Multimedia Appendix 1 [file cardio_v7i1e45137_app1.docx]

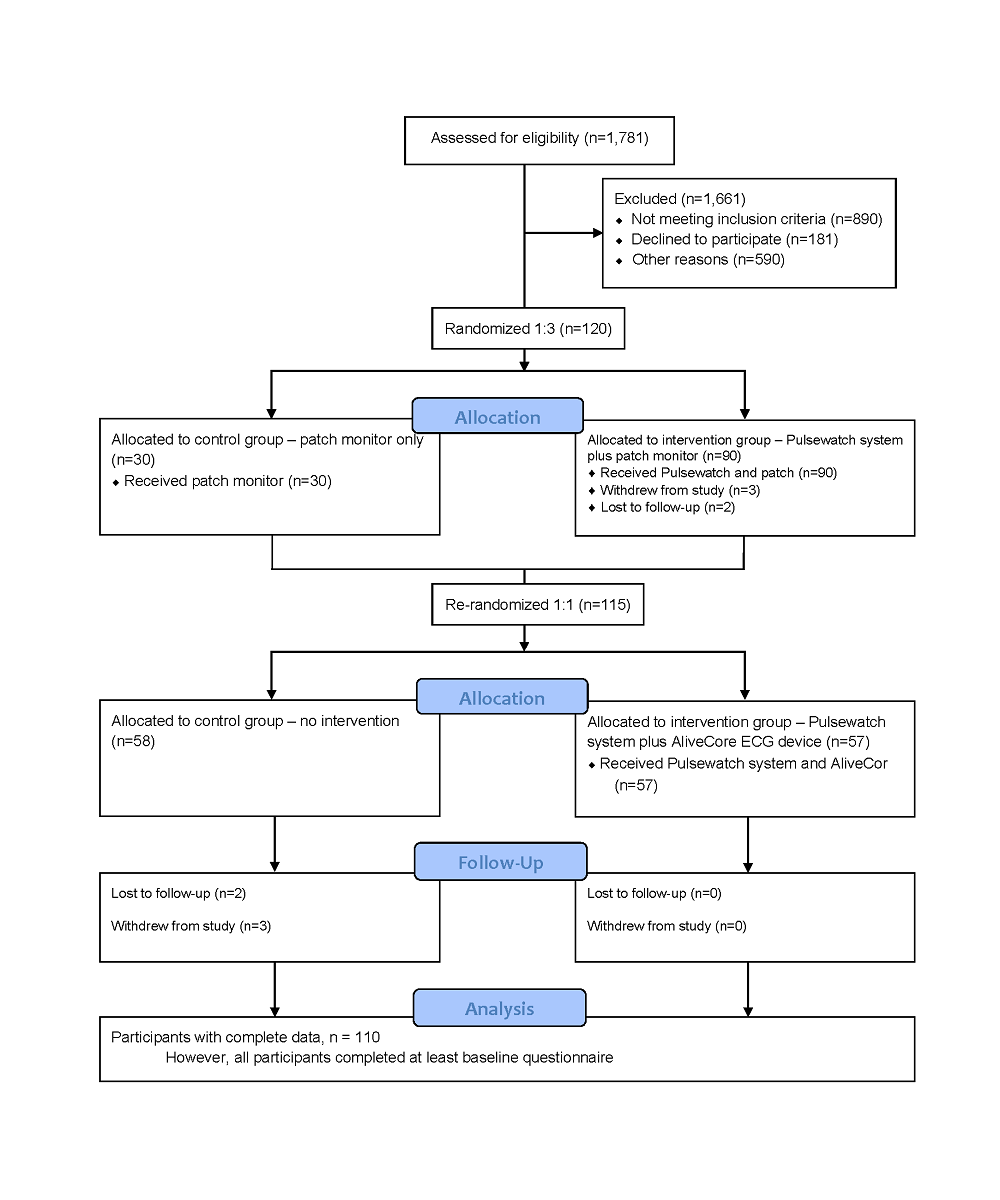

Supplement: Multimedia Appendix 2 [file cardio_v7i1e45137_app2.png]
